# mdm-miR828 Participates in the Feedback Loop to Regulate Anthocyanin Accumulation in Apple Peel

**DOI:** 10.3389/fpls.2020.608109

**Published:** 2020-12-02

**Authors:** Bo Zhang, Hui-Juan Yang, Ya-Zhou Yang, Zhen-Zhen Zhu, Ya-Nan Li, Dong Qu, Zheng-Yang Zhao

**Affiliations:** ^1^ State Key Laboratory of Crop Stress Biology for Arid Areas, College of Horticulture, Northwest A&F University, Yangling, China; ^2^ Apple Engineering and Technology Research Center of Shaanxi Province, Northwest A&F University, Yangling, China; ^3^ Shaanxi Key Laboratory Bio-resources, College of Bioscience and Engineering, Shaanxi University of Technology, Hanzhong, China

**Keywords:** apple, anthocyanin, mdm-miR828, *MdMYB1*, high temperature

## Abstract

Anthocyanins are responsible for the red pigmentation in the peel of apple (*Malus × domestica* Borkh.) fruit. Relatively few studies have investigated anthocyanins at the posttranscriptional level. MicroRNAs play an important role in plant growth and development by regulating gene expression at the posttranscriptional level. In this study, mdm-miR828 showed a relatively low expression level during the rapid fruit coloration period. However, the mdm-miR828 expression level increased in the late fruit coloration stage. Overexpression of mdm-miR828 inhibited anthocyanin synthesis in apple and *Arabidopsis*. Dual-luciferase and yeast one-hybrid assays showed that MdMYB1 is capable of binding to the promoter of mdm-MIR828b to promote its expression. The results indicate that mdm-miR828 is involved in a feedback regulatory mechanism associated with anthocyanin accumulation in apple. In addition, mdm-miR828 is involved in the inhibition of anthocyanin accumulation in response to high temperature.

## Introduction

Apple (*Malus* × *domestica* Borkh.) is an economically important fruit crop that is cultivated worldwide. The peel color is an important phenotypic trait of apple fruit, which largely determines the commodity value of the fruit. Anthocyanins, a diverse group of secondary metabolites in plants is responsible for the red coloration in apple peel (Takos et al., 2006). In addition, anthocyanins may be nutritionally and medically beneficial to human health (Chaves-Silva et al., 2018). The mechanism of anthocyanin synthesis has been elucidated in many plant species, for example, petunia (*Petunia hybrida*; de Vetten et al., 1997; Quattrocchio et al., 1999; Spelt et al., 2000; Albert et al., 2011, 2014), *Arabidopsis thaliana* (Borevitz et al., 2000; Gonzalez et al., 2008; Petroni and Tonelli, 2011), grape (*Vitis vinifera*; Kobayashi et al., 2004; Hichri et al., 2010; Matus et al., 2010), nectarine (*Prunus persica*; Ravaglia et al., 2013), and strawberry (*Fragaria* × *ananassa*; Aharoni et al., 2001; Lin-Wang et al., 2010; Medina-Puche et al., 2014). Anthocyanins are synthesized *via* the flavonoid metabolism pathway (Kim et al., 2003). Many enzymes participate in this pathway, such as chalcone synthase (CHS), flavanone 3-hydroxylase (F3H), dihydroflavonol 4-reductase (DFR), anthocyanidin synthase (ANS), and flavonoid 3-*O*-glycosyltransferase (UFGT; Holton and Cornish, 1995; Shirley et al., 1995; Kim et al., 2003). These enzymes are coregulated by the MYB-bHLH-WD40 (MBW) transcription factor complex (Goff et al., 1992; Baudry et al., 2004; Lloyd et al., 2017). Three alleles *MdMYB10*, *MdMYB1*, and *MdMYBA* that regulate anthocyanin synthesis in apple have been identified. These transcription factors directly bind to the promoter of anthocyanin biosynthesis genes to promote expression of the latter (Takos et al., 2006; Ban et al., 2007; Espley et al., 2007). For example, a cold-induced bHLH transcription factor, MdbHLH3, directly binds to the promoter of the structural gene *MdUFGT* and that of *MdDFR* to activate their expression and also binds to the promoter of the regulatory gene *MdMYB1* to promote its expression (Xie et al., 2012).

MicroRNA (miRNA) is a type of endogenous noncoding single-stranded small RNA in eukaryotes that predominantly guides posttranscriptional gene-silencing activities (Cuperus et al., 2011). The miRNA genes are first transcribed into primary transcripts in the nucleus and then processed into miRNA precursors (pre-miRNAs/MIRNA; Song et al., 2010). Subsequently, these pre-miRNAs are processed to form mature miRNAs (Voinnet, 2009). In addition to miRNAs, small interfering RNAs (siRNAs) are synthesized in plants (Rajagopalan et al., 2006), of which one is *trans*-acting small interfering RNA (ta-siRNA). The miRNA cleaves *Trans-Acting SiRNA* (*TAS*) loci transcripts, and then with the aid of RNA-dependent RNA polymerase 6 (RDR6) and suppressor of gene silencing 3 (SGS3), double-stranded RNA is synthesized and is cleaved by the DCL4 enzyme to produce ta-siRNAs. Its mechanism of action is similar to that of miRNA, that is, cleavage of the target transcript to interfere with gene expression (Allen et al., 2005).

MiRNAs are involved in the regulation of anthocyanin synthesis in plants (Liu et al., 2016; Sun et al., 2017). In *Arabidopsis*, SPL9, the target gene of miR156, competes with a bHLH transcription factor to bind to the MYB75 (PAP1) transcription factor, thereby disrupting the structure of the MBW protein complex and, ultimately, negatively regulating anthocyanin synthesis (Gou et al., 2011). *Arabidopsis* overexpressing miR778 accumulates higher amounts of anthocyanins than the wild type (WT) under phosphorus deficiency (Wang et al., 2015). The miR858 induced by elongated hypocotyl5 (HY5) targets the anthocyanin inhibitor MYBL2 to promote anthocyanin accumulation in *Arabidopsis* (Wang et al., 2016b). The miR828 was the first miRNA identified to negatively regulate anthocyanin accumulation. In *Arabidopsis*, miR828 targets the positive regulator of anthocyanin synthesis MYB113 and cleaves *TAS4* to produce TAS4-siR81(−), whereas siR81(−) targets the anthocyanin-related transcription factors PAP1, PAP2, and MYB113 (Rajagopalan et al., 2006). Xia et al. (2012) conducted sequencing of sRNAs from the leaf, root, flower, and fruit of “Golden Delicious” apple and observed that mdm-miR828 cleaves *MdTAS4* to derive mdm-siR81(−), and it was predicted that mdm-siR81(−) might target *MdbHLH3*. RNA gel blot analysis showed that mdm-miR828 predominantly accumulates in flowers. However, “Golden Delicious” is an apple cultivar with yellow fruit peel. It is unclear whether mdm-miR828 and mdm-siR81(−) are involved in the coloration of fruit with red peel, and the relationship between mdm-miR828 and MdMYB1 is uncertain.

In the present study, we investigated the expression pattern of mdm-miR828 during fruit coloration in the red-peeled apple “Starkrimson Delicious.” Overexpression of mdm-miR828 inhibited anthocyanin accumulation in the fruit peel, and heterologous overexpression of mdm-miR828 inhibited anthocyanin accumulation in *Arabidopsis*. MdMYB1 induced expression of mdm-miR828, which indicated that a feedback regulatory mechanism controlled the anthocyanin content in the peel. In addition, mdm-miR828 may be involved in the inhibition of anthocyanin accumulation in response to high temperature.

## Materials and Methods

### Plant Materials

Plants of apple “Starkrimson Delicious” were grown at the Apple Experimental Farm of Northwest A&F University, Shaanxi Province, China. Fruit were sampled at 110, 116, 122, 128, 134, 140, and 146 days after blooming. Nine fruit were selected at each time point with three fruit considered as one biological replicate. The peel was excised with a fruit peeler, immediately frozen with liquid nitrogen, and stored at −80°C until use. Transient transformation and high-temperature treatment were applied to fruit that were wrapped in a paper bag (Hongtai, Shanxi, China) at 45 days after blooming and harvested at 120 days after blooming.

### Determination of Anthocyanin Content

The total anthocyanin content of the fruit was determined using the method described by Xie et al. (2012). A sample (0.5 g) of peel was extracted in 5 ml of 1% (vol/vol) HCl-methanol and incubated in the dark at 4°C for 24 h. After centrifugation at 13,000 × *g* for 5 min, the absorbance of the upper liquid was measured at 530, 620, and 650 nm. The anthocyanin content was calculated using the formula OD = (*A*_530_ − *A*_620_) − 0.1(*A*_650_ − *A*_620_). One unit of anthocyanin content was expressed as a change of 0.1 OD (unit × 10^3^ g^−1^ fresh weight). Measurement of cyanidin 3-galactoside content was performed as described previously (Liu et al., 2013). A high-performance liquid chromatograph (Waters, Milford, MA, United States) was used for all analyses. Cyanidin 3-galactoside standard (Sigma Chemical, St Louis, MO, United States) was used. Separation of cyanidin 3-galactoside was accomplished on a C18 column (5-μm internal diameter, 250 × 4.6 mm; Waters). The anthocyanin content of *Arabidopsis* was determined using the method of Wang et al. (2016b).

### RNA Extraction and Quantitative Real-Time Polymerase Chain Reaction Analysis

Total RNA was extracted using TRIzol RNA Plant Plus Reagent (Tiangen, Beijing, China) and then treated with DNase I (TaKaRa, Dalian, China) to remove genomic DNA contamination. The RNA was reverse-transcribed with the PrimeScript™ RT Reagent Kit (TaKaRa, Dalian, China). The reverse transcription of mdm-miR828 and mdm-siR81(−) used specific stem-loop primers (Supplementary Table S1; Chen et al., 2005), and that of the internal reference gene *MdU6* used gene-specific primers (Supplementary Table S1). Quantitative real-time polymerase chain reaction (RT-qPCR) analysis was conducted using SYBR® Premix Ex Taq™ II (TaKaRa, Dalian, China) on an ABI StepOnePlus™ Real-Time PCR System (Applied Biosystems, Waltham, MA, United States). Poly (A) polymerase (PAP) RT-qPCR was implemented using the miRcute Plus miRNA First-Strand cDNA Kit (Tiangen, Beijing, China) and miRcute Plus miRNA qPCR Kit (Tiangen, Beijing, China). For normalization, 5S ribosomal RNA was used. *MdActin* was used to normalize the coding genes. The 2^−ΔΔCT^ method was used to calculate the relative expression level. Quantitative primer sequences used are listed in Supplementary Table S1.

### RNA Ligase-Mediated 5'-RACE

To verify the cleavage of *MdTAS4* by mdm-miR828 and that of *MdbHLH3* by mdm-siR81(−), RNA ligase-mediated rapid amplification of cDNA ends (RLM-RACE) experiments was performed using the SMARTer® RACE 5'/3' Kit (TaKaRa, Dalian, China). First, total RNA was reverse-transcribed using SMARTScribe™ reverse transcriptase, and the 5' adaptor was attached. Next, 5' universal primers, 3' gene-specific primers, and the cDNA were used for PCR amplification. Gel extraction, in-fusion cloning, and sequencing were then performed.

### Vector Construction and Genetic Transformation

The coding sequence (CDS) of *MdbHLH3* was cloned from the cDNA derived from “Starkrimson Delicious” fruit peel. The sequence of Md-pri-miR828b (comprising the Md-MIR828b sequence and approximately 100-bp upstream and downstream) was amplified from DNA extracted from “Starkrimson Delicious” peel. The sequences were inserted into the pCAMBIA2301 vector to form *35S:MdbHLH3* and *35S:mdm-miR828*. The primer sequences used are listed in Supplementary Table S1. The constructed vector was transformed into *Agrobacterium tumefaciens* strain GV3101.

*Agrobacterium*-mediated transformation followed the methods of Yang et al. (2019) and Tian et al. (2015). *Agrobacterium* cells harboring the overexpression vector were grown, collected, and resuspended in *Agrobacterium* infiltration buffer (200 mM acetosyringone, 10 mM MES, and 10 mM MgCl_2_) to OD = 1.2. The suspension was incubated at room temperature for 4 h. The sampled bagged covered apples were infiltrated with *Agrobacterium* carrying *35S:MdbHLH3*, *35S:mdm-miR828*, or the pCAMBIA2301 empty vector and then placed at room temperature in the dark for 24 h and transferred to an incubator maintained at 22°C illuminated with white light (200 μmol m^−2^ s^−1^) for 3 days.

The *35S:mdm-miR828* vector was used to transform *A. thaliana* using the floral-dip method (Clough and Bent, 1998). T3 generation of 35S:miR828 transformed *Arabidopsis* was used for the experiment.

### Transient Dual-Luciferase Assay

A transient dual-luciferase assay was performed using tobacco (*Nicotiana benthamiana*) leaves. The mdm-MIR828b promoter was amplified and cloned into the pGreenII 0800-LUC vector. The CDS of *MdMYB1* was amplified and cloned into the pGreenII 62-SK vector. The constructs were transformed into *Agrobacterium* strain GV3101 carrying the pSoup vector. *Agrobacterium* cells harboring the different vectors were cultured, collected, and resuspended in infiltration buffer (200 mM acetosyringone, 10 mM MES, and 10 mM MgCl_2_). After incubation for 2 h, tobacco leaves were injected with the cell suspension. After initial incubation for 24 h in the dark, the leaves were incubated under light (200 μmol m^−2^ s^−1^) with a 16 h/8 h (light/dark) photoperiod at 25/22°C (light/dark) for 3 days. Leaf discs were punched from tobacco leaves of similar size, and a microplate reader (Infinite M200 PRO, Tecan, Switzerland) was used to determine the firefly luciferase and *Renilla* luciferase activities using the Dual-Glo® Luciferase Assay System (Promega, Madison, WI, United States) in accordance with the manufacturer’s instructions.

### Yeast One-Hybrid Assay

The CDS of MdMYB1 was inserted into the pGADT7 vector to generate the recombinant vector MdMYB1-AD, and the mdm-MIR828b promoter fragment was inserted into the pHIS2.1 vector to generate the recombinant vector *proMIR828b-HIS*. Yeast (*Saccharomyces cerevisiae*) strain Y187 cells carrying the *proMIR828b-HIS* vector were cultured in −T−H screening medium supplemented with different concentrations of 3-amino-1,2,4-triazole (3-AT). The MdMYB1-AD and *proMIR828b-HIS* vectors were cotransformed into yeast Y187 cells, which were then cultured in −T−H−L selective medium containing an optimal concentration of 3-AT.

### High-Temperature Treatment

Bagged covered fruit of “Starkrimson Delicious” apple were harvested at 120 days after blooming. The harvested fruit were placed in an incubator at 35°C under white light (200 μmol m^−2^ s^−1^). The control was placed in an incubator at 23°C and under white light (200 μmol m^−2^ s^−1^). The fruit were sampled after 12, 24, and 48 h for analysis of anthocyanin content and gene expression. Three biological replicates, consisting of three fruit per replicate, were sampled at each time point.

## Results

### Anthocyanin Content and mdm-miR828 Expression During Apple Coloration

**Figure 1:**
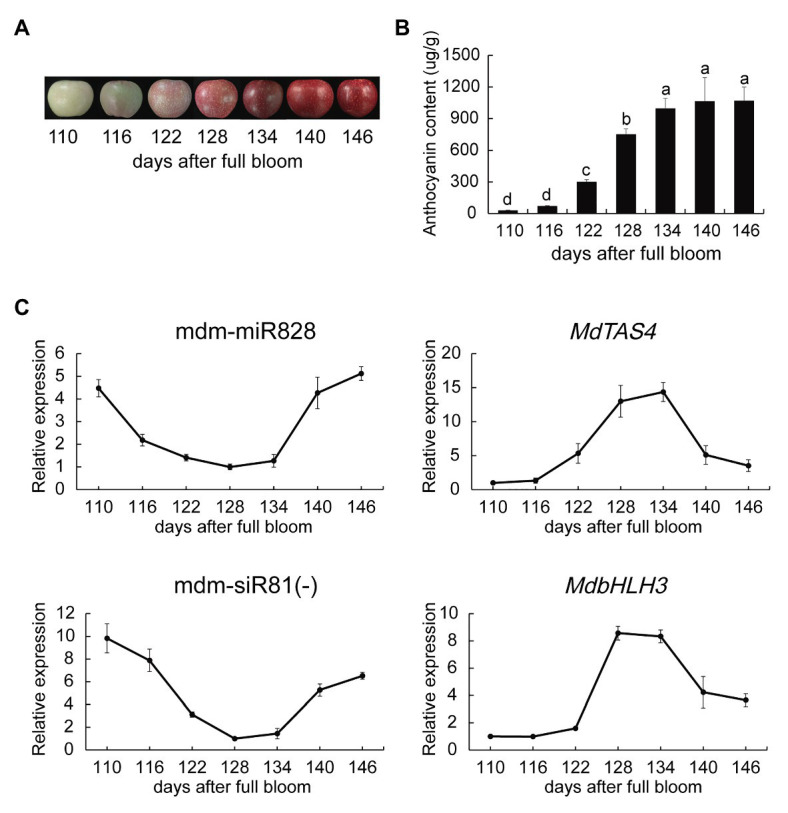
Anthocyanin content and mdm-miR828 expression during coloration of apple “Starkrimson Delicious” fruit. **(A)** Change in peel color during coloration. **(B)** Peel anthocyanin content during coloration. **(C)** Relative expression levels of mdm-miR828, mdm-siR81(−), and anthocyanin biosynthesis-related genes. The expression levels of mdm-miR828 and mdm-siR81(−) were detected by the stem-loop method. *MdU6* was used as an internal reference. Error bars represent the standard deviation of three biological replicates. Different letters above the bars indicate a significant difference (*p* < 0.05; one-way ANOVA and LSD test).

We determined the total anthocyanin content in the peel of “Starkrimson Delicious” fruit without covering at 110, 116, 122, 128, 134, and 146 days after blooming. No change in anthocyanin content was observed from 110 to 116 days, whereas anthocyanins accumulated rapidly in the peel from 116 to 134 days (the rapid coloration period). The anthocyanin content stabilized from 134 to 146 days (the late fruit coloration stage; Figures 1A,B). The content of cyanidin 3-galactoside, which accounted for more than 94% of the total anthocyanin content, showed a similar trend (Supplementary Figure S1).

We used the stem-loop method to detect the expression of mdm-miR828 in the peel. Expression of mdm-miR828 decreased rapidly from 110 to 116 days and showed a relatively low expression level during the period of rapid anthocyanin synthesis (116–134 days). However, mdm-miR828 expression increased from 134 to 146 days (Figure 1C). The PAP RT-qPCR method yielded similar results (Supplementary Figure S2). In addition, we measured the expression levels of *MdTAS4*, mdm-siR81(−), and *MdbHLH3* (Figure 1C). The expression level of *MdTAS4* increased from 116 to 134 days and thereafter decreased. That of mdm-siR81(−) declined rapidly from 116 to 128 days, remained relatively low from 128 to 134 days, and increased after 134 days. The expression level of *MdbHLH3* increased sharply from 122 to 128 days and thereafter decreased from 134 to 146 days. The expression level of anthocyanin structural genes (*MdCHS*, *MdDFR*, *MdANS*, and *MdUFGT*) increased from 116 to 134 days and then decreased to varying degrees (Supplementary Figure S2).

### Overexpression of mdm-miR828 Inhibits Anthocyanin Accumulation in Apples

**Figure 2:**
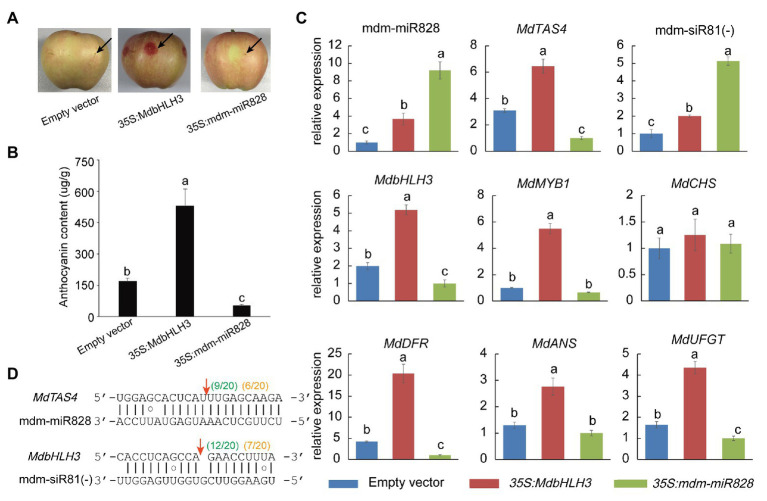
Transient expression of mdm-miR828 in apple “Starkrimson Delicious” fruit. **(A)** Overexpression of mdm-miR828 inhibits anthocyanin accumulation in the peel. The black arrows represent the injection sites. **(B)** Anthocyanin content in the peel of fruit overexpressing mdm-miR828 or *MdbHLH3*. **(C)** Overexpression of mdm-miR828 and *MdbHLH3* affects the expression level of anthocyanin biosynthesis-related genes in the peel. **(D)** The binding positions of mdm-miR828 and mdm-siR81(−) in the target genes *MdTAS4* and *MdbHLH3*. Green numbers and orange numbers in parentheses indicate the proportion of 5'-RACE clones at the sites where the target gene is cleaved by mdm-miR828 and mdm-siR81(−) in mdm-miR828-overexpressing peel and mixed peels (harvested at 134, 140, and 146 days after blooming), respectively. Error bars represent the standard deviation of three biological replicates. Different letters above the bars indicate a significant difference (*p* < 0.05, one-way ANOVA and LSD test).

To confirm that mdm-miR828 is involved in anthocyanin biosynthesis in the peel, we generated the construct *35S:mdm-miR828* and injected the fruit peel of “Starkrimson Delicious” with *Agrobacterium* carrying this construct. Transient overexpression of mdm-miR828 inhibited anthocyanin accumulation around the injection site, whereas no reduction in anthocyanin content was observed after injection of *Agrobacterium* containing the empty vector. Thus, mdm-miR828 was indicated to negatively regulate anthocyanin accumulation in apple. In addition, overexpression of *MdbHLH3* significantly promoted accumulation of anthocyanins in the peel (Figures 2A,B).

The expression of anthocyanin synthesis-related genes was further analyzed (Figure 2C). The expression levels of *MdbHLH3*, *MdbMYB1*, *MdDFR*, *MdANS*, and *MdUFGT* increased to varying degrees compared with those of the control (empty vector) in the peel of fruit overexpressing *MdbHLH3*. In addition, mdm-miR828, *MdTAS4*, and mdm-siR81(−) expression levels were higher than those of the control. In the peel of fruit overexpressing mdm-miR828, both mdm-miR828 and mdm-siR81(−) were up-regulated compared with those of the control (empty vector), whereas the expression levels of *MdTAS4*, *MdbHLH3*, *MdDFR*, *MdANS*, and *MdUFGT* decreased compared with those of the control.

The RLM-RACE procedure was applied using the peel of fruit overexpressing mdm-miR828 to determine the cleavage site of mdm-miR828 on the *MdTAS4* transcript and that of mdm-siR81(−) on the *MdbHLH3* transcript. Some predicted 5' free ends of *MdTAS4* and *MdbHLH3* (between the 10th and 11th nucleotides of the miRNA binding site to the target gene) were detected (Figure 2D). In addition, we mixed samples of peels (collected at 134, 140, and 146 days) during the coloration period and performed the RACE experiment. The cleavage sites of mdm-miR828 and mdm-siR81(−) on the target genes *MdTAS4* and *MdbHLH3* were also detected.

### Overexpression of mdm-miR828 in *Arabidopsis* Inhibits Anthocyanin Accumulation

**Figure 3:**
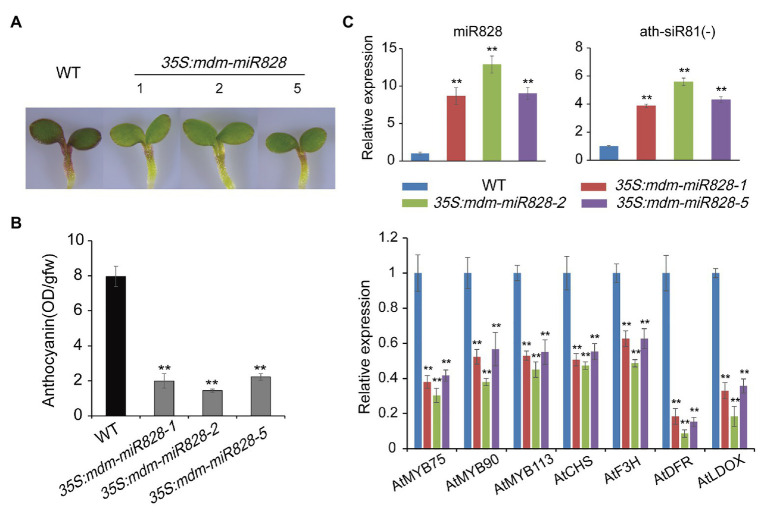
Overexpression of mdm-miR828 inhibits anthocyanin accumulation in *Arabidopsis* seedlings. **(A)** Phenotypes of mdm-miR828-overexpressing and wild-type *Arabidopsis* grown on half-strength Murashige and Skoog medium supplemented with 2% sucrose for 4 days. 35S:mdm-miR828-1, 35S:mdm-miR828-2, and 35S:mdm-miR828-5 represent three transgenic lines. **(B)** Anthocyanin content in mdm-miR828-overexpressing and wild-type *Arabidopsis*. **(C)** Relative expression levels assessed by qRT-PCR analysis showed that mdm-miR828 overexpression affects expression of anthocyanin-related genes in *Arabidopsis*. Error bars represent the standard deviation of three biological replicates. ^**^p < 0.01 (Student *t*-test).

To confirm the function of mdm-miR828 in anthocyanin biosynthesis, we generated mdm-miR828-overexpressing *Arabidopsis* transformants. Sucrose can induce the accumulation of anthocyanins in *Arabidopsis* seedlings (Teng et al., 2005). Therefore, seeds of WT and mdm-miR828-overexpression lines were cultured on half-strength Murashige and Skoog solid medium supplemented with 2% sucrose. The anthocyanin content was determined after culture for 4 days. The anthocyanin content in the mdm-miR828-overexpression lines was significantly lower than that of the WT. This result indicated that mdm-miR828 negatively regulated anthocyanin synthesis in *Arabidopsis* (Figures 3A,B).

Expression of siR81(−) in the mdm-miR828-overexpression *Arabidopsis* seedlings was increased compared with that of the WT. The expression levels of anthocyanin biosynthesis-related regulatory genes (*AtMYB75*, *AtMYB90*, and *AtMYB113*) and structural genes (*AtCHS*, *AtDFR*, *AtF3H*, and *AtLDOX*) were reduced compared with those of the WT. These results suggested that mdm-miR828 and ath-miR828 performed similar functions and negatively regulated anthocyanin synthesis in *Arabidopsis* (Figure 3C).

### MdMYB1 Promotes Expression of mdm-miR828

**Figure 4:**
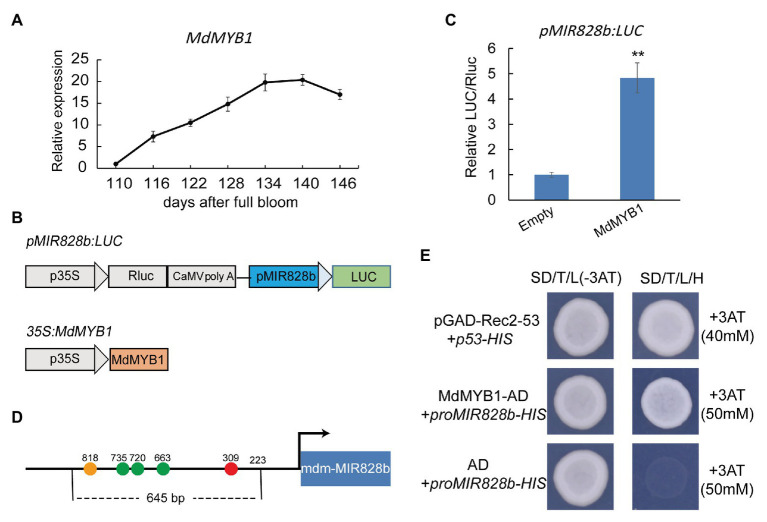
MdMYB1 promotes mdm-MIR828b expression by directly binding to its promoter. **(A)** Expression pattern of *MdMYB1* during the coloration period of apple “Starkrimson Delicious” fruit. **(B)** Schematic representation of the construct used for the dual-luciferase assay. **(C)** Relative LUC/REN ratio. REN, *Renilla* luciferase activity; LUC, firefly luciferase activity. Error bars represent the standard deviation of three biological replicates. ^**^p < 0.01 (Student *t*-test). **(D)** Schematic diagram of the mdm-MIR828b promoter structure. Circles represent the potential MYB transcription factor recognition sites: orange (TAACCA), green (CAACCA), and red (TAACTG). The number represents the distance (bp) from the transcription start site. **(E)** Yeast one-hybrid assay for binding of the mdm-MIR828b promoter and MdMYB1. pGAD-Rec2-53 and p53-HIS were used as positive controls.

An autoregulatory feedback loop operates in *Arabidopsis*, and up-regulated expression of PAP1/MYB75 can activate miR828 expression (Hsieh et al., 2009; Luo et al., 2012). As a consequence, miR828 generates a greater amount of siR81(−) by directing the cleavage of *TAS4* transcripts, thereby enhancing the cleavage of the target genes *PAP1/MYB75*, *PAP2/MYB90*, and *MYB113* (Hsieh et al., 2009; Luo et al., 2012). In the present experiment, the expression levels of mdm-miR828 and mdm-siR81(−) increased in the late stage of apple coloration (134–146 days; Figure 1C), whereas *MdMYB1* maintained a high level of expression in the late stage of coloration (Figure 4A). We speculated that MdMYB1 directly regulates expression of mdm-miR828. To test this hypothesis, we used a dual-luciferase assay to assess the correlation between MdMYB1 and the mdm-MIR828 promoter. First, we detected the expression levels of mdm-MIR828a (or pre–mdm-miR828a) and mdm-MIR828b. Expression of mdm-MIR828a was essentially undetectable, whereas the expression level of mdm-MIR828b was increased in the late fruit coloration stage (134–146 days; Supplementary Figure S3). Therefore, we cloned the 1,514-bp sequence of the mdm-MIR828b promoter and ligated it into the pGreenII 0800-LUC vector to form the fusion vector *pMIR828b:LUC* (Figure 4B). We then used *Agrobacterium* carrying *pMIR828b:LUC* and those harboring *35S:MdMYB1* to cotransform *N. benthamiana* leaves. Compared with the empty plasmid (pGreenII 62-SK) control, transient overexpression of *MdMYB1* significantly up-regulated LUC activity driven by the mdm-MIR828b promoter (Figure 4C). This result indicated that MdMYB1 promoted the expression of mdm-miR828. Using the PlantCARE database, we detected multiple potential MYB transcription factor recognition sites in the 645-bp segment upstream of the mdm-MIR828b promoter (Figure 4D). A yeast one-hybrid assay showed that MdMYB1 is able to bind to this segment in the mdm-MIR828b promoter (Figure 4E).

### mdm-miR828 Participates in the Process of High-Temperature Inhibition of Anthocyanin Accumulation

**Figure 5:**
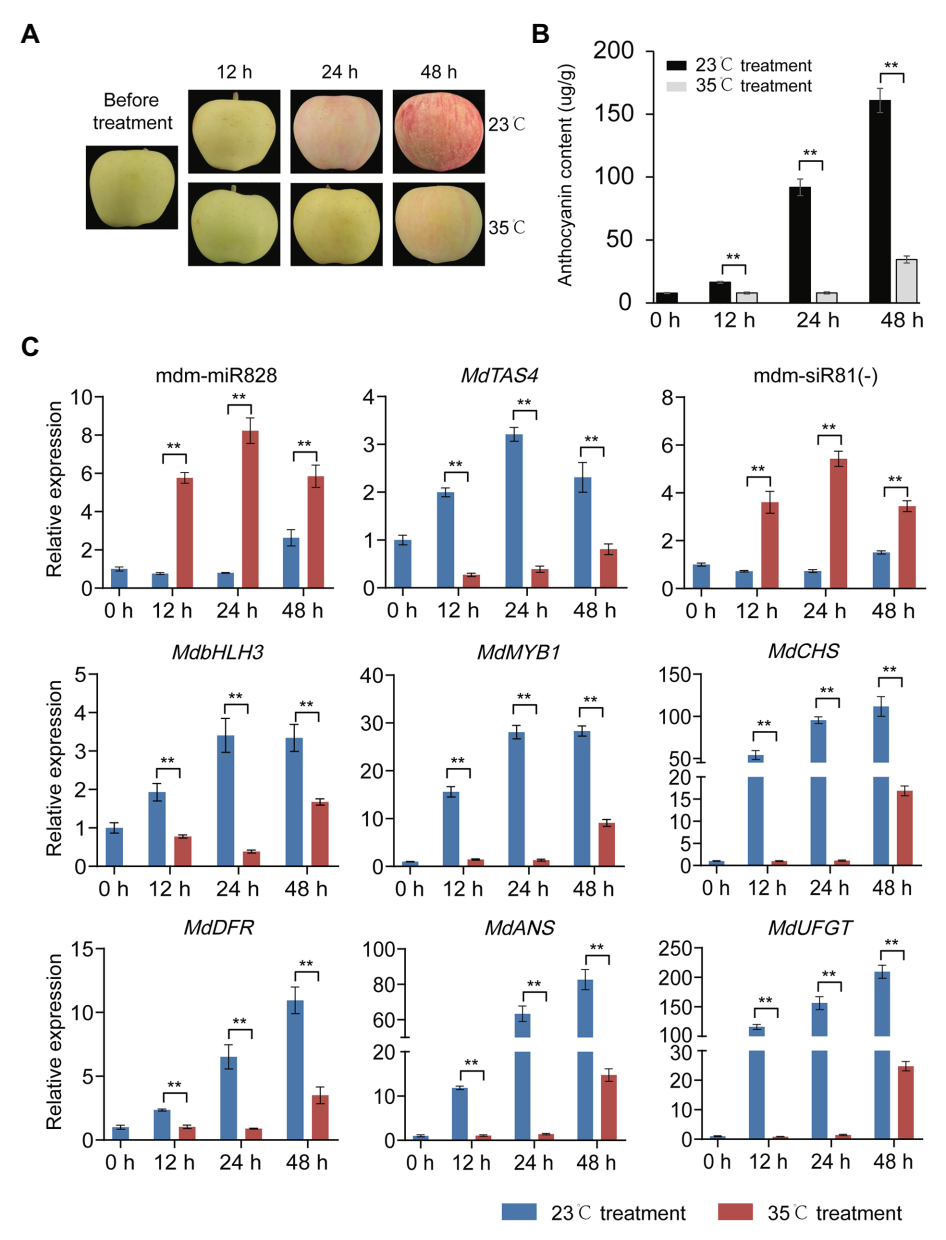
High temperature inhibits anthocyanin accumulation in apple peel. **(A)** Change in color of “Starkrimson Delicious” peel under high temperature. **(B)** Anthocyanin content in the peel under high temperature. **(C)** Relative expression level of mdm-miR828, mdm-siR81(−), and anthocyanin-related genes (*MdTAS4*, *MdbHLH3*, *MdbMYB1*, *MdCHS*, *MdDFR*, *MdANS*, and *MdUFGT*) under high temperature. Error bars represent the standard deviation of three biological replicates. ^**^p < 0.01 (Student *t*-test).

High temperature can inhibit anthocyanin accumulation in apple (Fang et al., 2019). As a potential negative regulator of anthocyanin accumulation, it is not clear whether mdm-miR828 is involved in the inhibition of anthocyanin accumulation in response to high temperature. We placed previously bagged covered “Starkrimson Delicious” fruit in an incubator maintained at either 35 or 23°C (the control). Under continuous light for 24 h, apple anthocyanin accumulation was reduced at 35°C compared with that of the control group at 23°C, and the difference was more obvious at 48 h (Figures 5A,B). The expression level of mdm-miR828 was elevated under high temperature compared with that of the control. The expression level of mdm-siR81(−) also increased in response to high temperature, whereas the expression levels of the anthocyanin synthesis-related genes *MdTAS4*, *MdbHLH3*, *MdMYB1*, *MdCHS*, *MdDFR*, *MdANS*, and *MdUFGT* were significantly reduced under high temperature compared with those of the control (Figure 5C). These results suggested that mdm-miR828 may be involved in the process of high-temperature inhibition of anthocyanin accumulation.

## Discussion

Anthocyanins are an important subclass of flavonoids that not only provide rich pigmentation to flowers and fruits, but also have anticancer, antioxidation, antiaging, and other beneficial properties for human health (Venancio et al., 2017). In addition, anthocyanins are involved in resistance to diverse biotic and abiotic stresses (Guo et al., 2008; Kovinich et al., 2015). The synthesis of anthocyanins in plants is strictly regulated by sophisticated regulatory networks. Previous studies of anthocyanin synthesis have predominantly focused on regulation at the transcriptional level, whereas research at the posttranscriptional level is relatively rare. In plants, miRNAs play a vital regulatory role at the posttranscriptional level largely through cleavage of target genes. Therefore, identification of miRNAs associated with fruit coloration is important for improvement of fruit quality.

In plants, the MBW complex, composed of an MYB transcription factor, bHLH transcription factor, and WD40 protein, is highly conserved and coordinately regulates structural genes of the anthocyanin synthesis pathway (Gonzalez et al., 2008). A deletion mutation of *TT8* (a member of the bHLH transcription factor family) in *Arabidopsis* causes a severe decrease in the expression level of *DFR* and *BAN* genes, which causes a change in the seed coat color to yellow (Nesi et al., 2000). In apples, the transcription factor MdbHLH3 induced by low temperature can interact with MdMYB1 and promotes the expression of *MdDFR* and *MdUFGT* structural anthocyanin biosynthesis genes. In addition, MdbHLH3 may bind to the promoter of the regulatory gene *MdMYB1* to promote its expression (Xie et al., 2012). In the present study, during the period of rapid synthesis of anthocyanins, *MdbHLH3* was significantly increased, and overexpression of *MdbHLH3* in the fruit promoted anthocyanin accumulation. These findings are consistent with previous results (Xie et al., 2012) and reveal that *MdbHLH3* is essential for anthocyanin synthesis. The *TAS4* gene is conserved in many dicotyledonous plants. Luo et al. (2012) compared *TAS4* sequences from *A. thaliana*, *Theobroma cacao*, *Euphorbia esula*, *Actinidia chinensis*, *V. vinifera*, and *Mimulus guttatus* and observed that the binding site of miR828 is conserved. In the present study, RACE proved that mdm-miR828 directs cleavage of the *MdTAS4* transcript between the 10th and 11th nucleotides, which indicated that mdm-miR828-mediated cleavage of *MdTAS4* transcripts is conserved in apple. In *Arabidopsis*, ath-siR81(−) can target the anthocyanin-related transcription factors PAP1/MYB75, PAP2/MYB90, and MYB113. However, there is no sequence complement between mdm-siR81(−) and the anthocyanin-related transcription factor MdMYB1 in apples. Interestingly, mdm-siR81(−) is complementary to *MdbHLH3* sequence, whereas mdm-siR81(−) and *MdbHLH3* show an inverse expression profile to each other. In addition, the RACE experiment demonstrated that mdm-siR81(−)-directed cleavage of *MdbHLH3* transcripts occurs in the peel of apple fruit.

Regulation of anthocyanin synthesis by miRNAs has been observed in many plant species, but the effects of miRNAs on anthocyanins differ. Inhibition of miR156 expression in *Arabidopsis* results in increased expression of the target gene *SPL9* and thus inhibits accumulation of anthocyanins (Gou et al., 2011; Cui et al., 2014). In contrast, overexpression of mdm-miR156a in apple causes decreased expression of the *SPL2-like* and *SPL33* genes and inhibition of anthocyanin accumulation (Yang et al., 2019). Overexpression of miR858 in *Arabidopsis* targets the anthocyanin inhibitor MYBL2 to promote anthocyanin accumulation (Wang et al., 2016b). However, miR858 in tomato plays a negative role in the biosynthesis of anthocyanins; blockage of miR858 binding increases *SlMYB7-like* transcript levels and ultimately promotes accumulation of anthocyanins (Jia et al., 2015). In *Arabidopsis*, siR81(−) derived from *TAS4* and miR828 together target the positive regulatory genes *PAP1*, *PAP2*, and *MYB113* for anthocyanin synthesis (Rajagopalan et al., 2006). Overexpression of miR828 reduces the accumulation of anthocyanins in *Arabidopsis* (Yang et al., 2013). However, miR828 targets the anthocyanin inhibitor MYB114 in grape to positively regulate anthocyanin accumulation (Tirumalai et al., 2019). In the present study, the mdm-miR828 expression level was relatively low during the rapid fruit coloration period (116 to 134 days), and mdm-siR81(−) expression was also relatively low. In addition, overexpression of mdm-miR828 in apple fruit inhibited anthocyanin synthesis, and overexpression of mdm-miR828 in *Arabidopsis* inhibited anthocyanin accumulation. These results indicated that mdm-miR828 negatively regulated anthocyanin synthesis in apple.

In the *Arabidopsis* mutant *pap1-D*, in which *PAP1* is strongly up-regulated, high quantities of anthocyanins accumulate, but the expression levels of ath-miR828 and ath-siR81(−) also increase (Hsieh et al., 2009). Phosphorus deficiency, nitrogen deficiency, and exogenous sugars treatment can increase the expression of the PAP1/MYB75 and PAP2/MYB90 MYB transcription factors, which in turn activates the expression of anthocyanin structural genes and increases anthocyanin synthesis in *Arabidopsis*. In these plants with high anthocyanin content, the expression levels of ath-miR828 and ath-siR81(−) increased, which induced negative feedback regulation of AtMYB75, AtMYB90, and AtMYB113 expression. These results indicate that a feedback regulatory mechanism in *Arabidopsis* may control anthocyanin accumulation (Hsieh et al., 2009; Luo et al., 2012). In the current study, the anthocyanin tended to remain stable in the late stage of fruit coloration, whereas *MdMYB1* maintained a high level of expression from 134 to 140 days after blooming, and at this stage, the expression level of mdm-miR828 began to increase. The dual-luciferase and yeast one-hybrid assays showed that MdMYB1 is capable of binding to the promoter of mdm-MIR828b to induce its expression. These results indicated that a feedback regulatory mechanism operates in apple. In addition, *MdMYB1* and mdm-miR828 expression levels were increased in fruit overexpressing *MdbHLH3*, which suggested that the feedback pathway was activated.

In addition to sucrose, light (Takos et al., 2006) and low temperature (Xie et al., 2012; Gaiotti et al., 2018) promote anthocyanin synthesis. However, high temperature inhibits biosynthesis of anthocyanins in apple (Wang et al., 2016a). In the present experiment, anthocyanin accumulation under high temperature (35°C) was significantly inhibited compared with that of the control (23°C). The expression levels of mdm-miR828 and mdm-siR81(−) were higher under high temperature compared with those of the control, whereas the expression level of *MdbHLH3* was reduced under high temperature compared with that of the control. These findings indicated that mdm-miR828 may be involved in the process of inhibiting anthocyanin accumulation under high temperature.

**Figure 6:**
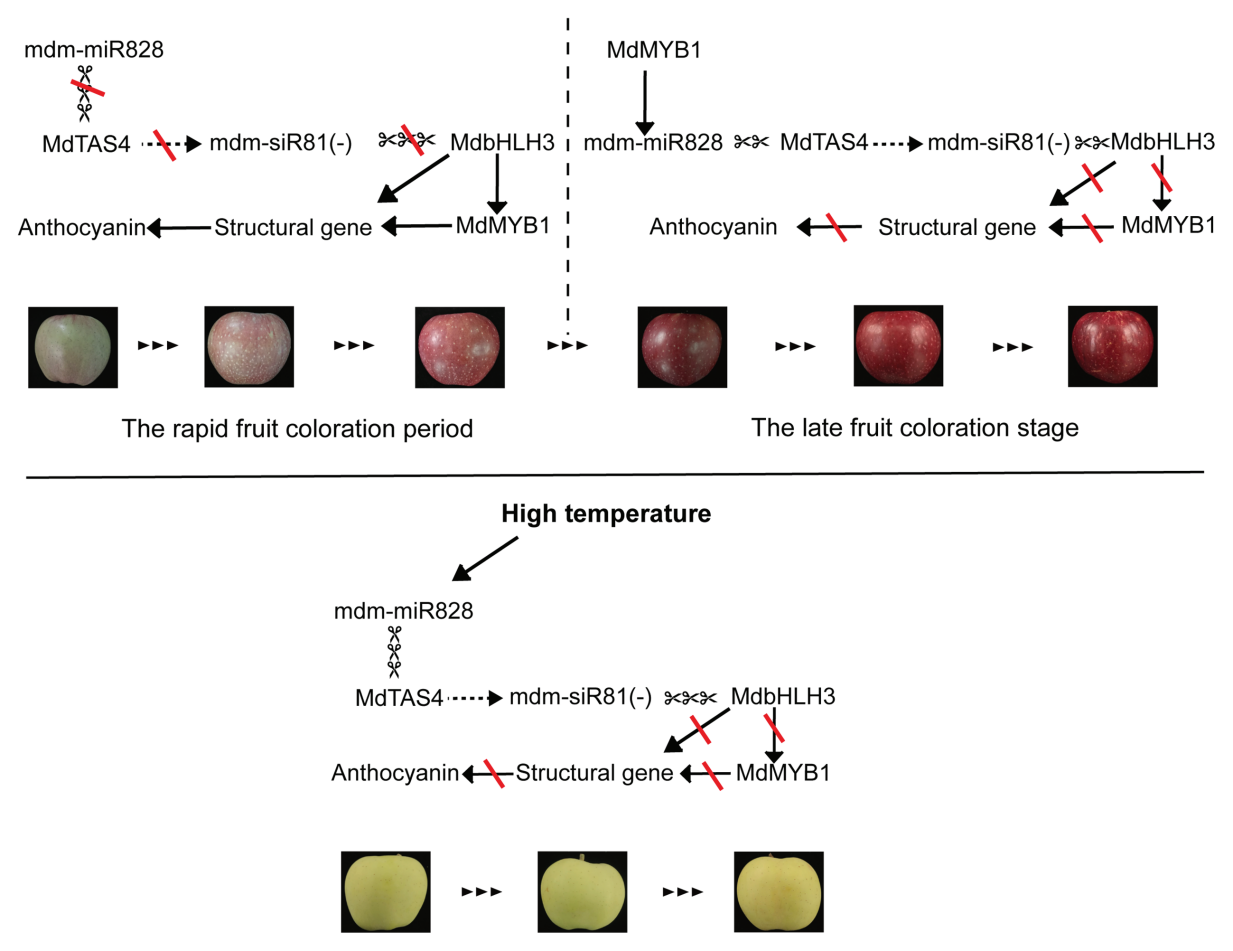
Proposed model for mdm-miR828 participation in a feedback regulation pathway to balance anthocyanin accumulation in the peel of apple fruit. A solid arrow indicates activation, a dashed arrow indicates derivation, scissors indicate cleavage, and red lines indicate closure.

Based on the present results, we propose a model for mdm-miR828 participation in a feedback regulatory mechanism to balance anthocyanin accumulation in the peel of apple fruit (Figure 6). In this model, mdm-miR828 regulates the expression of *MdbHLH3* by cleaving *MdTAS4* to derive mdm-siR81(−). During the period of rapid fruit coloration, the expression level of mdm-miR828 is low, and *MdbHLH3* and *MdMYB1* are expressed in large quantities, which promotes the rapid synthesis of anthocyanins. In the late fruit coloration stage, the high-level expression of MdMYB1, which binds to the promoter of mdm-MIR828b, promotes the expression of mdm-miR828, which in turn inhibits the expression of *MdbHLH3* so that anthocyanins do not accumulate indefinitely. In addition, high temperature can also induce the expression of mdm-miR828 and mdm-siR81(−), and then the highly expressed mdm-siR81(−) cleaves *MdbHLH3* to prevent the accumulation of anthocyanins.

## Data Availability Statement

The original contributions presented in the study are included in the article/Supplementary Material, further inquiries can be directed to the corresponding author.

## Author Contributions

BZ, H-JY, and Z-YZ conceived the original screening and research plans. BZ, Y-ZY, Z-ZZ, and Y-NL supervised the experiments. BZ, H-JY, and DQ analyzed the data. BZ wrote the article. H-JY revised the manuscript. All authors contributed to the article and approved the submitted version.

### Conflict of Interest

The authors declare that the research was conducted in the absence of any commercial or financial relationships that could be construed as a potential conflict of interest.
