# Supplementary material for: mdm-miR828 Participates in the Feedback Loop to Regulate Anthocyanin Accumulation in Apple Peel
Source: Front Plant Sci. 2020 Dec 2;11:608109. doi: 10.3389/fpls.2020.608109 (PMC7774908; doi:10.3389/fpls.2020.608109)
Supplement: Supplementary file 1 [file Data_Sheet_1.docx]

Supplementary Material


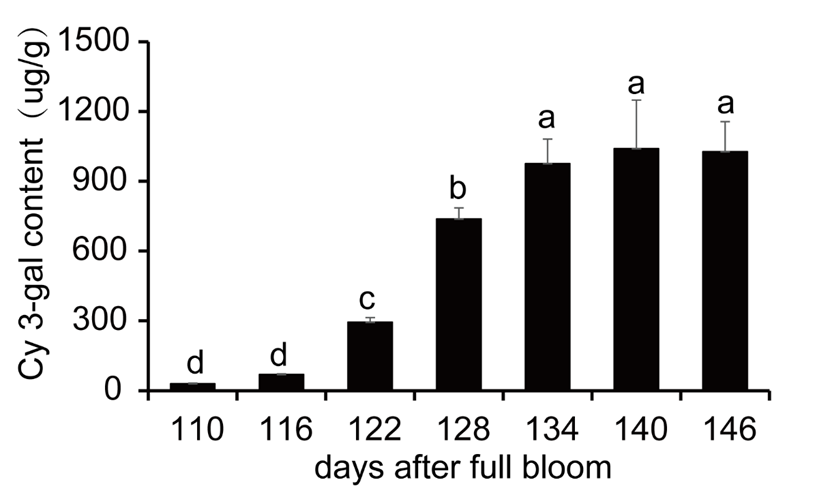


**Figure S1.** Cyanidin 3-galactoside content during coloration of apple 'Starkrimson Delicious' fruit. Error bars represent the standard deviation of three biological replicates. Different letters above the bars indicate a significant difference (*P* < 0.05; one-way ANOVA and LSD test).


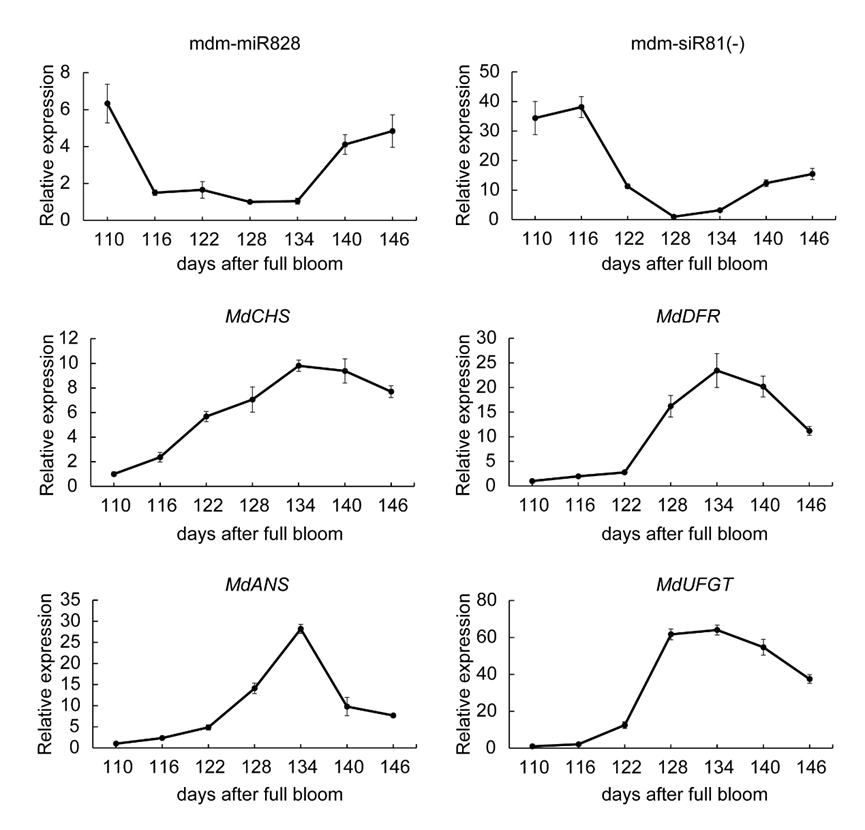


**Figure S2.** Relative expression levels of mdm-miR828, mdm-siR81(-), and anthocyanin structural genes. The expression levels of mdm-miR828 and mdm-siR81(-) were detected by the poly(A) polymerase (PAP) RT-qPCR method. Apple 5s rRNA was used as an internal reference. Error bars represent the standard deviation of three biological replicates.


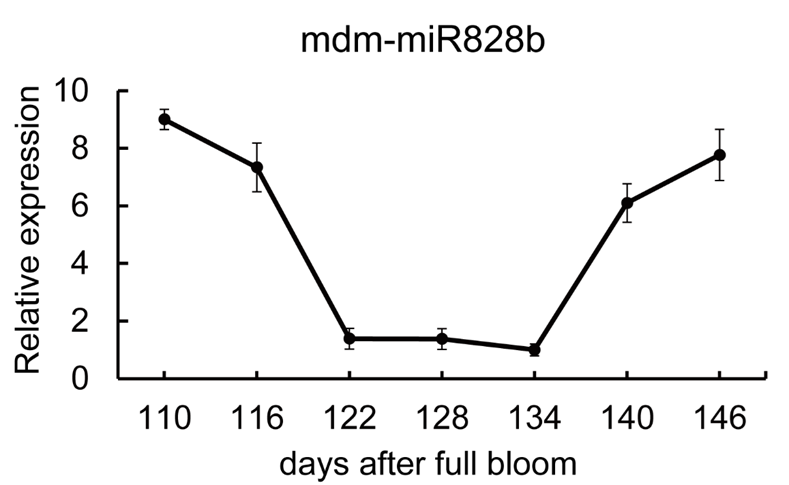


**Figure S3.** Expression pattern of mdm-MIR828b during the coloration period of apple 'Starkrimson Delicious' fruit. Error bars represent the standard deviation of three biological replicates.
